# Comparing chemical transfection, electroporation, and lentiviral vector transduction to achieve optimal transfection conditions in the Vero cell line

**DOI:** 10.1186/s12860-024-00511-x

**Published:** 2024-05-13

**Authors:** Parisa Jamour, Abbas Jamali, Arash Ghalyanchi Langeroudi, Behrouz Ebadi Sharafabad, Asghar Abdoli

**Affiliations:** 1 Department of Hepatitis and HIV, Pasteur Institute of Iran, Tehran, Iran; 2 Student Research Committee, Pasteur Institute of Iran, Tehran, Iran; 3 Department of Influenza and Other Respiratory Viruses, Pasteur Institute of Iran, Tehran, Iran; 4 Department of Microbiology and Immunology, Faculty of Veterinary Medicine, University of Tehran, Tehran, Iran; 5 Department of Pharmaceutical Biotechnology, Faculty of Pharmacy, Tabriz University of Medical Science, Tabriz, Iran

**Keywords:** Vero cell line, Chemical transfection reagents, Transfection efficiency, Electroporation, TurboFect, Lentivirus

## Abstract

**Background:**

Transfection is an important analytical method for studying gene expression in the cellular environment. There are some barriers to efficient DNA transfection in host cells, including circumventing the plasma membrane, escaping endosomal compartmentalization, autophagy, immune sensing pathways, and translocating the nuclear envelope. Therefore, it would be very useful to introduce an optimum transfection approach to achieve a high transfection efficiency in the Vero cell line. The aim of this study was to compare various transfection techniques and introduce a highly efficient method for gene delivery in Vero cells.

**Methods:**

In the current study, three transfection methods were used, including chemical transfection, electroporation, and lentiviral vector transduction, to obtain the optimum transfection conditions in the Vero cell line. Vero cells were cultured and transfected with chemical transfection reagents, electroporation, or HIV-1-based lentivectors under different experimental conditions. Transfection efficiency was assessed using flow cytometry and fluorescence microscopy to detect GFP-positive cells.

**Results:**

Among the tested methods, TurboFect™ chemical transfection exhibited the highest efficiency. Optimal transfection conditions were achieved using 1 µg DNA and 4 µL TurboFect™ in 6 × 10^4^ Vero cells.

**Conclusion:**

TurboFect™, a cationic polymer transfection reagent, demonstrated superior transfection efficiency in Vero cells compared with electroporation and lentivirus particles, and is the optimal choice for chemical transfection in the Vero cell line.

## Background

Transfection is a widely used laboratory technique that involves the introduction of exogenous nucleic acids into cells to study gene function and products in a cellular context [1]. The success of transfection depends on several factors, including the quality of the nucleic acids, duration of the transfection process, transfection reagent used, and specific cell lines employed. Localization of the introduced nucleic acids within the cell is crucial for determining the efficacy of the procedure, and the degradation of nucleic acids by cellular nucleases such as deoxyribonuclease II (DNase II) poses a significant challenge [2]. As nucleic acids alone cannot permeate the cell membrane, it is necessary to use suitable carriers for transportation. To investigate the effect of DNase II on transfection efficiency, we modulated its expression levels in transfected cells. Their findings demonstrated a negative correlation between elevated DNase II levels and the effectiveness of non-viral DNA delivery vectors, providing definitive evidence that DNase II constitutes a significant impediment to successful transfection [3]. Various strategies have been developed for nucleic acid delivery, including physical, chemical, and viral transduction [4].

The Vero cell line is the most widely used continuous cell line for the production of viral vectors and vaccines. Historically, this was the first cell line approved by the WHO for the production of human vaccines. The Vero cell line, derived from the kidney epithelial cells of an African green monkey, is a continuously passaged cell line that can be propagated for a long period. It is susceptible to various viruses and lacks a responsive cellular pathway that is triggered by interferon expression. Therefore, it has great potential as a viable option for the production of viral vaccines. These cells have found applications in various research fields such as virology and toxicology [5]. Therefore, regulatory bodies of the World Health Organization have recommended the use of the Vero cell line as the primary continuous cell line for manufacturing viral vaccines for human use [6].

Chemical transfection strategies involve the use of various substances, such as Ca2 + phosphatepolycation and dendrimers, to facilitate DNA transfer across cell membranes. Cationic polymers, such as poly-L-lysine, lipopolyamine, and polyamidoamine, have been studied as potential vehicles for delivering nucleic acids, showing efficient transfection without damaging the cellular membranes [7, 8].

Physical transfection methods include microinjection, optical, biolistic, and electroporation [9]. Electroporation is commonly used method in laboratories to obtain higher transfection efficiencies. By applying a transient high-pressure current pulse, cells undergo a process that results in the formation of nanometer-level micropores within their membranes. Consequently, this phenomenon enables the entry of genetic materials, proteins, and other biological molecules into cells. Electroporation offers numerous advantages, owing to its simplicity, rapidity, reproducibility, safety, and compatibility with various cell types. This method is particularly effective for transecting suspension culture cells, which are typically difficult to transfect. Several factors can influence both the electroporation transfection efficiency and post-transfection cell viability. Therefore, optimizing the transfection conditions is necessary to ensure the successful transfection of different cellular strains [10].

Virus-mediated transfection or transduction uses viral vectors to introduce specific nucleic acid sequences into host cells. Lentiviral transduction is highly efficient for delivering transgenes to mammalian cells, especially primary cells that are difficult to transfect. However, viral transduction carries the risk of cytotoxicity and viral infection. Optimizing transfection conditions is crucial for achieving high efficiency and consistency, because different cell strains have specific requirements. The heterogeneity of transfection efficiency among cell types emphasizes the need for comparative studies to determine the most effective method for each cell type [11]. Enhanced gene transfer techniques specific to Vero cells, commonly used in vaccine development and gene therapy, are essential for successful gene expression studies.

## Materials and methods

### Cell culture

The Vero cell line (purchased from the National Cell Bank of Iran, Tehran) was grown in Dulbecco’s modified Eagle’s medium (DMEM) (Gibco, Invitrogen, USA) supplemented with 10% heat-inactivated fetal bovine serum (Gibco, Invitrogen, USA) and antibiotics (0.1 mg/mL streptomycin and 100 U/mL penicillin) (Biosera). Cells were cultured at 37 °C in a humidified incubator with 5% CO2.

### Plasmid purification

The pCDH-CMV-MCS-EF1-CopGFP-T2A-Puro plasmid obtained from System Bioscience (CD-513B-1, Australia) was purified using the Miniprep plasmid kit (Qiagen, Germany). The DNA concentration was determined using a UV-visible spectrophotometer. Plasmids were stored at -20 °C for subsequent use.

### Chemical transfection method

#### Lipofection

Two commercially available transfection reagents, Lipofectamine™ 2000 (Invitrogen, USA) and TurboFect™ (Invitrogen), were used for transfection. Vero cells were seeded in 24-well plates at a density of 6 × 10^4^ cells/well and allowed to adhere overnight. To initiate transfection, varying concentrations of the plasmid (pCDH-CMV-MCS-EF1-CopGFP-T2A-Puro) ranging from 500 ng to 1 and 2 µg, along with different amounts of the transfection reagents (1–2 and 4 µL), were diluted in 100 µL of OptiMEM medium and incubated at room temperature for 30 min. Subsequently, the mixture was added dropwise to the wells and incubated at 37 °C for 4 h. Following this incubation period, the supernatant was replaced with a fresh medium containing 10% FBS and 1% antibiotics. After 72 h, flow cytometry was performed to quantify GFP-positive cells. All experiments were performed in triplicates.

### X-tremeGENE™ 9 transfection

X-tremeGENE™ 9 DNA transfection reagent (Sigma‒Aldrich, USA) is a proprietary blend of lipids and other organic components supplied in 80% ethanol. This reagent was used for transfection, following the established protocol. Specifically, 500 ng–1 µg and 2 µg of plasmid DNA, along with 1–2 and 4 µL of the transfection reagent, were added to the cultured cells. After a 72-h incubation period, the expression level was quantitatively assessed using flow cytometry. All experiments were performed in triplicates.

### PEI MAX® transfection

Commercially available linear polyethylenimines, PEI MAX® (Polysciences, USA) 40 kDa was used as transfection reagent [12]. For transfection each 24-well plate were seeded with Vero cells at 6 × 10^4^ cells/well. Cells were incubated at 37 °C in 5% CO2 for 24 h before transfection. Briefly, the amount of 500 ng–1 µg and 2 µg of plasmid DNA diluted in 50 µL of Opti-MEM was mixed with 1–2 and 4 µL of PEI MAX® (1 mg/ml stock) and incubated at room temperature for 20 min. Cells were rinsed twice with 1 ml of DMEM supplemented with 1% GlutaMax (no antibiotics), and the plasmid DNA: PEI mixture was added to the rinsed cells containing 500 µL of DMEM. After 4 h incubation at 37 C and 5% CO2, transfection medium was replaced with 1 ml of Complete DMEM medium. transfected cells incubation 48 h and the GFP-positive cells was determined by flow cytometry. All experiments were performed in triplicates.

### Physical transfection methods

#### Electroporation

Three different buffer types and voltage levels were used for electroporation. Initially, Vero cells were washed three times with ice-cold phosphate-buffered saline (PBS) and then counted at a concentration of 0.3 ml × 1000,000 cells/ml. The counted cells were then suspended in Ebuffer 1, which contained sodium chloride (140 mM) and disodium hydrogen phosphate (pH∼7.3). Subsequently, 5 µg of plasmid (pCDH-CMV-MCS-EF1-CopGFP-T2A-Puro) was mixed with the Vero cell suspension. Ebuffer 2, comprising optimum + HEPES-buffered saline (10 mM, pH∼7.3) and phosphate-buffered sucrose (272 mM, pH∼7.3), was prepared and mixed with the counted Vero cells along with 5 µg of plasmid. Similarly, Ebuffer 3, consisting of RPMI 1640, dipotassium phosphate (10 mM), magnesium chloride (1 mM), and sucrose (250 mM, pH∼7.3), was mixed with 5 µg of the plasmid. Electroporation was performed in a 4-mm gapped cuvette and immediately pulsed using a Gene Pulser Xcell (Bio-Rad, Germany) with the electric parameters set at 850 µF in a square wave. Three different voltage levels, i.e.,200, 300, and 400 V) were employed. The resistance was set to 100 Ω and the pulse time was 20 ms. The cells were promptly transferred to a 6-well plate and cultured in a complete medium containing 12% FBS and 1% antibiotics. After 72 h of electroporation, the cells were trypsinized, and GFP-positive cells were determined by flow cytometry analysis using the Partec Particle Analysis System. All experiments were performed in triplicates.

### Virus-mediated transfection methods

#### Lentiviral vector production and purification

The transfer vector, pCDH-CMV-MCS-EF1-CopGFP-T2A-Puro, encodes green fluorescent protein (GFP) using the cytomegalovirus (CMV) promoter. It also included puromycin as the selection marker. In the process of viral production, we used an envelope vector called pMD2G (Addgene,12,259), which expresses the VSV-G protein, and a packaging vector called psPAX2 (Addgene,12,260), which expresses Gag-pol and Tat. These vectors are based on HIV-1 lentivectors. To generate lentivirus particles, we co-transfected the three plasmids into 5 × 10^5^ HEK 293 Lenti-X cells cultured in 6-well plates. At 48 and 72 h post-transfection, culture supernatants were collected and filtered using a 0.22 μm filter. The filtered supernatant was then subjected to ultracentrifugation at 50,000 × g for 4 h to obtain viral pellets. These pellets were subsequently resuspended in high-glucose DMEM, mixed well, and incubated overnight in a shaker. Finally, the viral particles were stored at -80 °C for future use.

#### In vitro transduction and determination of the lentiviral vector titer

The transduction process involved serial dilutions of vector-containing suspensions of 6 × 10^4^ Vero cells that had been seeded in 24-well plates on the previous day. Each transduction included 8 µg/mL polybrene (Sigma-Aldrich) in the transducing inoculum. After 6 h, the medium was changed, and after 3 days, trypsinized cells were resuspended in PBS. Cell transduction was determined by analyzing GFP-positive cells using Partec flow cytometry (Partec, Particle Analysis System). The calculation of transduction titers involved determining the percentage of cells expressing GFP, with 100,000 cells used for each individual experiment (*n* ≥ 3). The virus titer was calculated using the formula TU/ml = (F × N × D)/V, where TU/ml represents the amount of functional viral particles (Transducing Units) in 1 ml of the stock solution. where F represents the percentage of GFP-positive cells (not exceeding 0.20% for reliable titer estimation), N is the number of cells at the time of transduction, D is the fold-dilution of the vector used for transduction, and volts is the volume (ml) of the diluted vector samples [13]. All experiments were performed in triplicates.

### Cell viability analysis

The viability of each transfection was measured using trypan blue exclusion and was expressed as a percentage of the initial number of cells.

### Flow cytometry assay analysis

The percentage of cells that exhibited fluorescence was determined. (Transfected cells) using a Partec PAS instrument (Germany). To recognize GFP-positive cells, untransfected cells (control) were used as GFP-negative controls to establish the gates. Consequently, the gated region was analyzed for green fluorescence (488–508 nm) using a Partec PAS instrument supplied with a 6 W argon laser tuned to 488 nm at an output power of 100 mW. The transfection efficiency was determined by calculating the percentage of cells showing GFP emission in the gated region. Transfected cells were imaged using an inverted fluorescence microscope to obtain GFP fluorescence images. (Olympus BX51, London, UK) with a 10× lens and digital images were captured using a digital camera. The samples were evaluated using FlowJo v10 software (Tree Star Inc., Ashland, OR, USA).

### Statistical analysis

All data are shown as mean ± SD. A two-way ANOVA was performed to compare the means among all groups. All statistical analyses were performed using GraphPad Prism version 9.0. Differences were considered to be statistically significant at *p* ≤ 0.05.

## Results

### Optimizing the transfection efficiency of chemical-based reagents in the Vero cell line

To determine the highest transfection efficiency by chemical transfection reagent in the Vero cell line, 0.5, 1, and 2 µg of purified pCDH plasmid with 1, 2, and 4 µL of chemical transfection reagents, including Lipofectamine™ 2000, TurboFect™, X-tremeGENE™ 9, and PEI MAX®, were transfected into Vero cells.

**Fig. 1:**
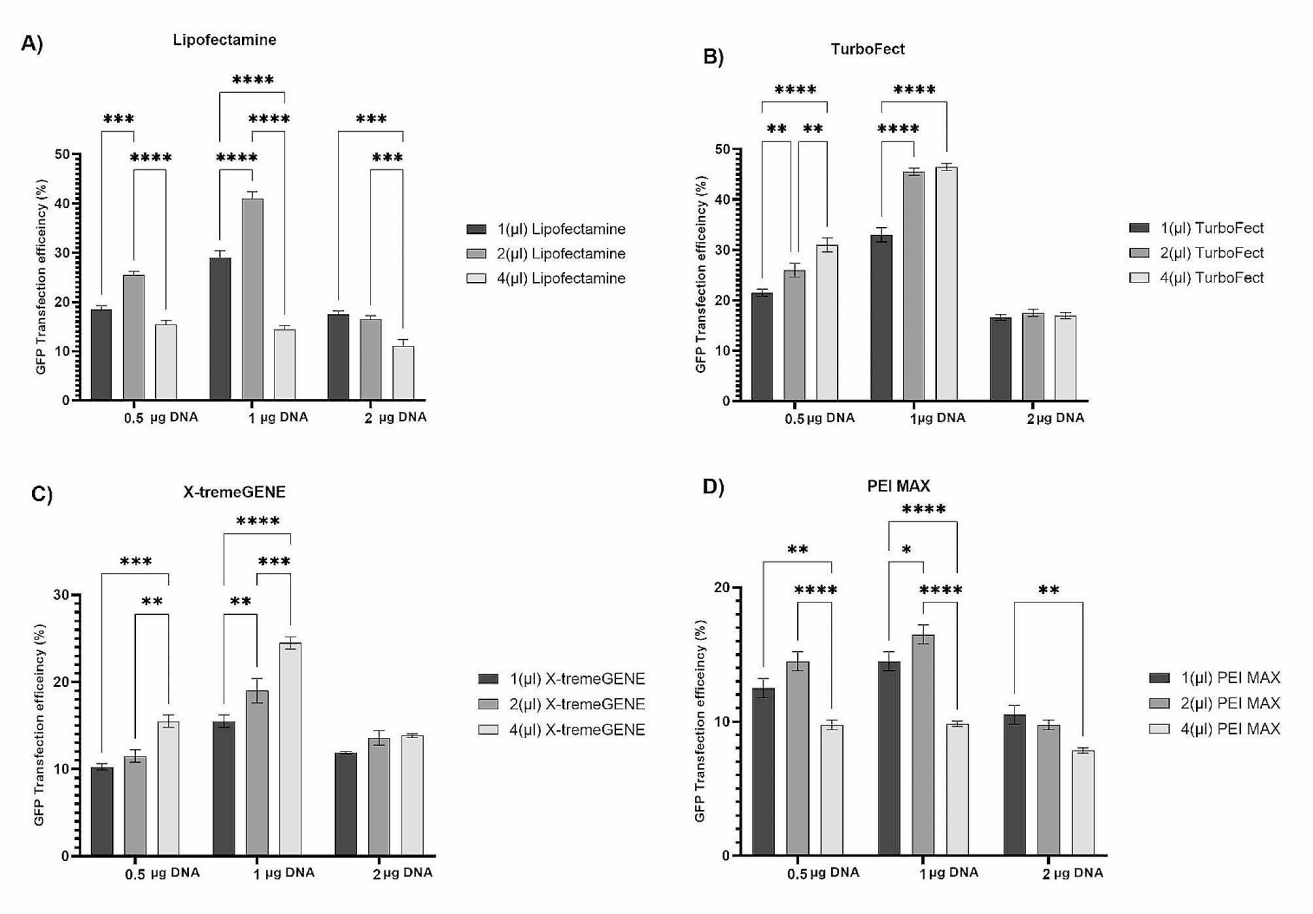
GFP transfection efficiency analysis chemical-based transfection reagents. Vero cell lines were subjected to transfection using Lipofectamine™ 2000 **(A)**, TurboFect™ **(B)**, X-tremeGENE™ 9 **(C)**, and PEI MAX® **(D)**. (*p* ≤ 0.05 *, *p* ≤ 0.01**, *p* ≤ 0.001***, *p* ≤ 0.0001****)

**Fig. 2:**
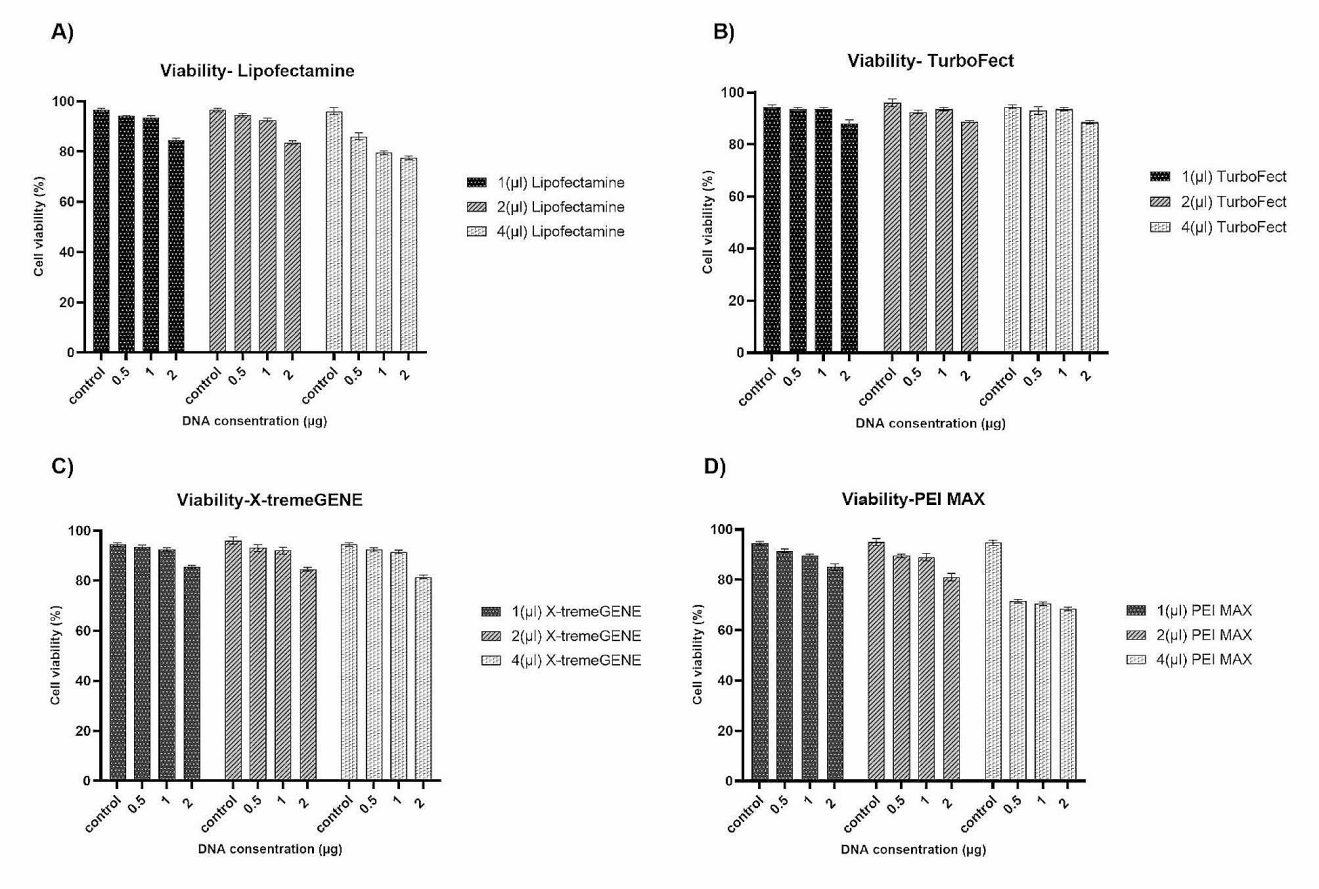
Cell viability analysis using chemical transfection reagents. Vero cell line viability was assessed using the chemical transfection reagents Lipofectamine™ 2000 **(A)**, TurboFect™ **(B)**, X-tremeGENE™ 9 **(C)**, and PEI MAX® **(D)**

The highest transfection efficiency was obtained with 1 µg plasmid to 2 µL Lipofectamine™ 2000 (Fig. 1A), and GFP transfection efficiency at this ratio was significantly different from the other ratios (*p* ≤ 0.05). As shown in Fig. 1A, there was a significant difference in other ratios of plasmid to Lipofectamine™ 2000, but the maximum transfection efficiency was observed at this ratio, with 41% GFP-positive cells. As shown in Fig. 2A, cell viability (∼93%) in this cell line using Lipofectamine™ 2000 was comparable, regardless of the ratio. No significant differences in cell viability were observed between control cells, which did not receive any DNA, and cells transfected with different reagent/DNA ratios (*p* ≤ 0.05).

The results in Fig. 1B show that TurboFect™ had the highest transfection efficiency at various DNA concentrations compared to the other transfection reagents. The highest transfection efficiency was achieved with a DNA/reagent ratio of 1 µg: 4 µL, which was significantly different from the other ratios. The presence of 46.5% GFP-positive cells with this DNA/reagent ratio confirmed that the concentrations of both reagents and DNA were suitable for the Vero cell line. Thus, TurboFect™ showed the best transfection efficiency compared with the other transfection reagents used in this experiment. As shown in Fig. 2B, the cell viability (∼94%) of this cell line using TurboFect™ was comparable, regardless of the ratio. No significant differences in cell viability were observed between control cells and cells transfected with different reagent/DNA ratios post-transfection (*p* ≤ 0.05, Fig. 2B).

According to the results obtained from Fig. 1c, X-tremeGENE™ 9 transfection reagent showed optimum transfection efficiency at a rate of 1 µg:4 µL of DNA/reagent. The estimated optimum number of GFP-positive cells was 24.5%, which was significantly different from the other DNA/reagent ratios (*p* ≤ 0.05, Fig. 1C). Cell viability (∼91%) was observed at this ratio, and there were no significant differences between these and the control cells (*p* ≤ 0.05, Fig. 2C).

The results obtained from the PEI MAX® transfection reagent showed high transfection efficiency in 1 µg:2 µL of DNA/reagent ratio, and GFP-positive cells were 16.5% (Fig. 1D). There was a significant difference compared with the DNA/reagent ratio (*p* ≤ 0.05). Cell viability (∼89%) was observed at this ratio and there were no significant differences between the control cells (*p* ≤ 0.05, Fig. 2D). A comparison of cell viability results in different transfection buffers showed that the percentage of Vero cell viability after transfection with the PEI MAX® reagent was lower than that of the control and other transfection buffers. This suggests that PEI MAX® may be more toxic to Vero cells than other buffers.

### Optimizing the electroporation conditions for different buffers and voltages in the Vero cell line

**Fig. 3:**
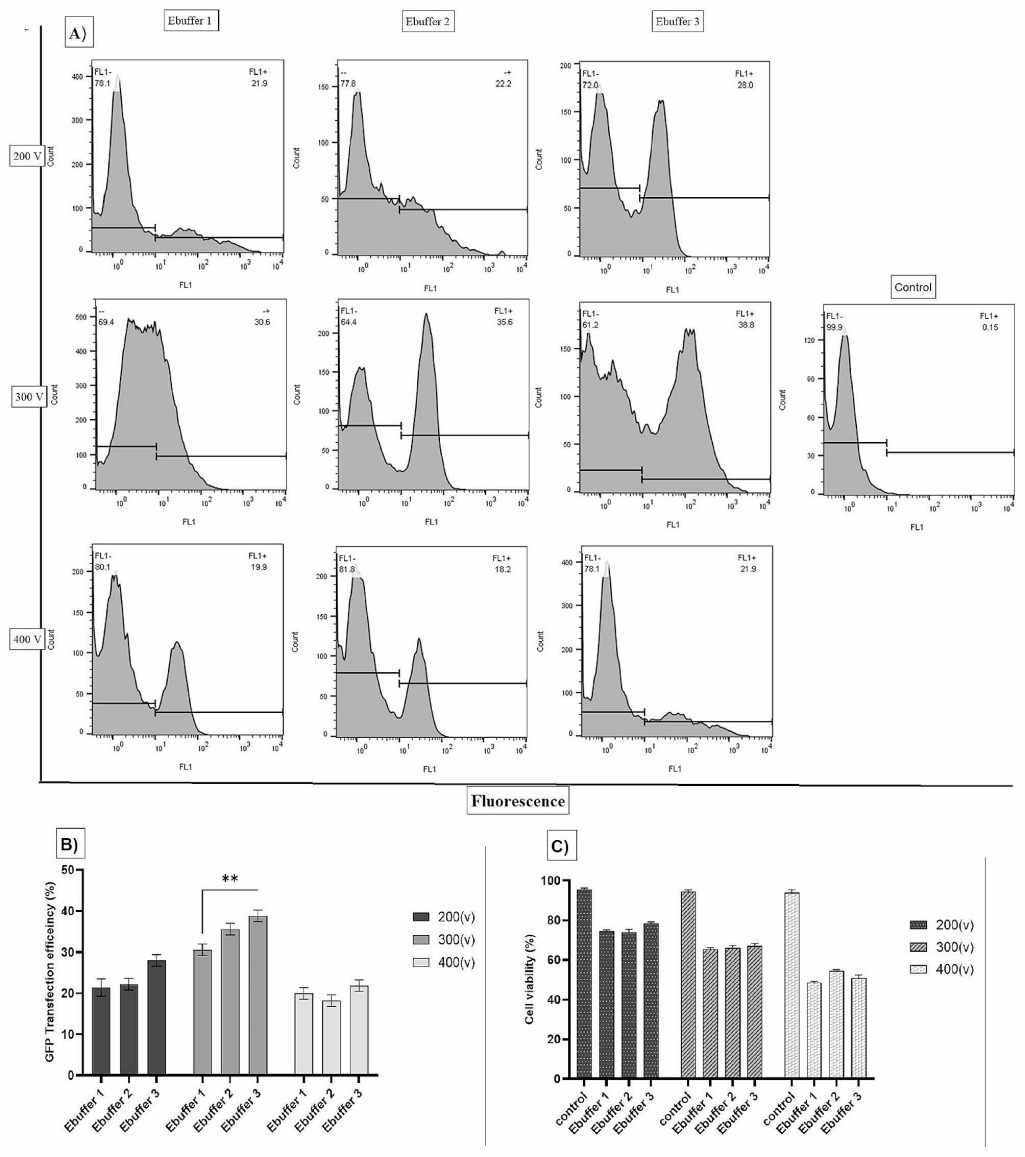
GFP transfection efficiency analysis of electroporation by flow cytometry demonstrated the expression of GFP at various voltages and Ebuffer in Vero cells by flow cytometry **(A)**, and analysis showed that there was a significant difference between Ebuffer 3 and Ebuffer 1 at 300 V **(B)** (*p* ≤ 0.01**). Comparison of cell viability in all groups with the control showed that the cell death rate was higher at 400 V than at the other voltages **(C)**

To demonstrate better electroporation conditions in the Vero cell line, three buffers were examined at three voltages. Figure 3A shows the results of flow cytometry of GFP-positive cells at voltages of 200, 300, and 400 v and Ebuffer1, 2, and 3 with various compositions and controls. The maximum transfection efficiency was observed in Ebuffer3 at 300 V (∼38.8%) (Fig. 3B), and the increase was statistically significant compared to that in Ebuffer1 at the same voltage (*p* ≤ 0.01). Therefore, the observed cell viability (∼67%) was compared to that of the control at this voltage (Fig. 3C). As shown in Fig. 3B, the increase in transfection efficiency at 300 V between buffers 1 and 3 was statistically significant. This increase may be attributed to the buffer composition, especially RPMI 1640, which was used for successful electroporation in Vero cells. As shown by the flow cytometry results, the transfection efficiency at 200 V was lower than that at 300 V, possibly because of insufficient voltage to open the plasma membrane pores and transfer DNA into the cell. Furthermore, analysis of cell viability data and flow cytometry results at 400 V showed that cell death was higher at this voltage than at other voltages, which may explain the lower percentage of GFP-positive cells and transfection efficiency.

### Optimizing lentiviral vector pseudotype production and transduction in Vero cell lines

**Fig. 4:**
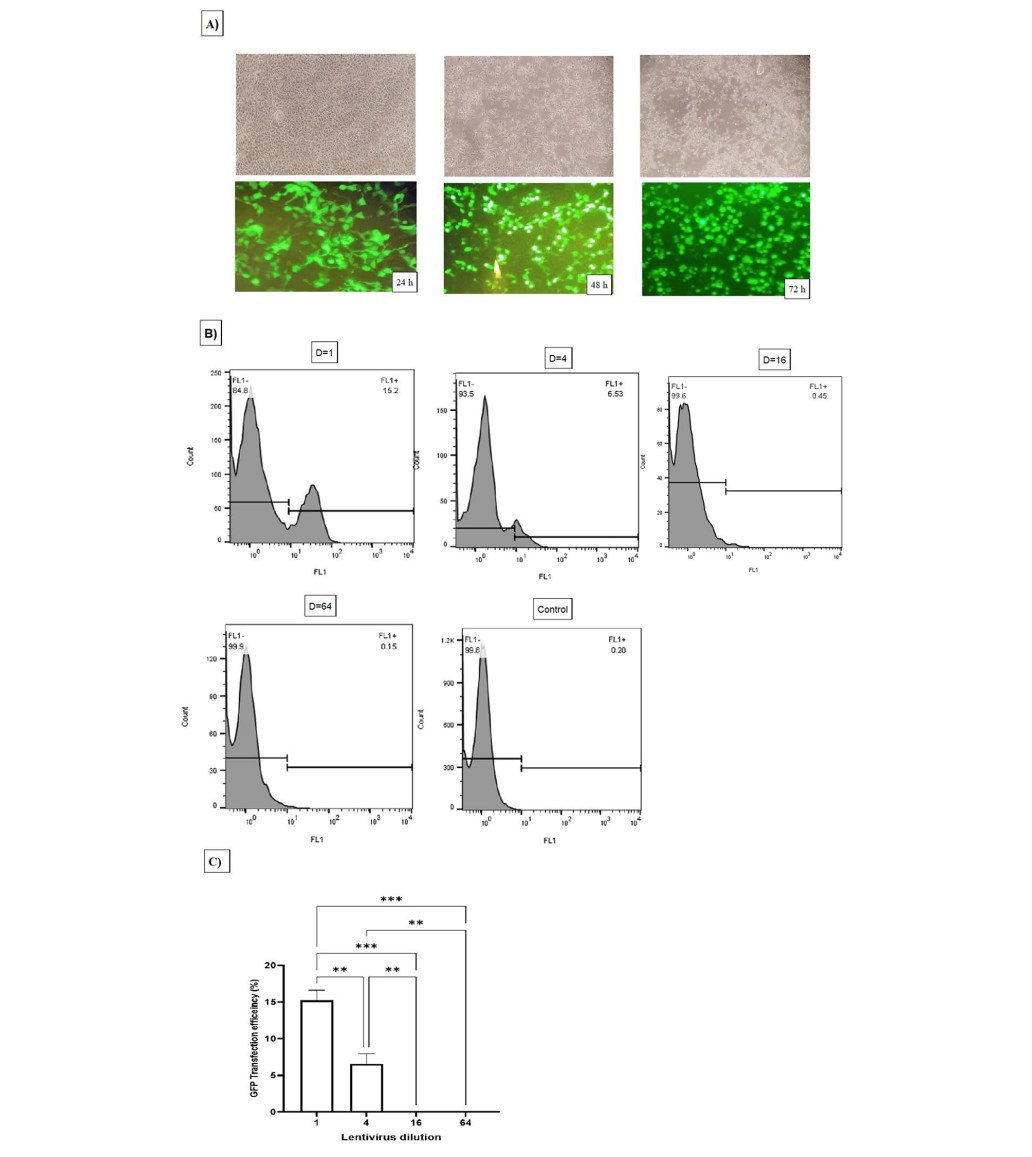
Lentiviral vector phenotype transduction in Vero cells. GFP expression in HEK293 Lenti-X cells at 24, 48, and 72 h post-transduction **(A).** Flow cytometry analysis of 1,4,16,64 virus dilution **(D)** transduced into Vero cells **(B)** and transfection efficiency in all dilutions in **(C)**

Lentivirus particles were produced in the HEK 293 Lenti-X cell line by co-transfection with three HIV-1-based lentivectors. The supernatant of the cells was collected at 48 and 72 h after transfection. Figure 4A shows GFP expression in cells at 24, 28, and 72 h after transfection. As shown in the images, more viral particles formed over time, which caused morphological changes in cells and the appearance of cytopathic effects of the virus on cells. Viral particles were ultracentrifuged for purification. The viruses were then transduced into Vero cells at dilutions (D) of 1, 4, 16, and 64, and the efficiency of transduction was measured by flow cytometry to detect GFP expression in the cells. Figure 4B shows the flow cytometry results for the different viral dilutions transduced into Vero cells. The highest transduction efficiency (∼15.2%) was observed at a dilution of one virus. The titer of the produced lentiviruses was calculated to be 1.9 × 10^5^ (TU/ml) using the formula described above and the percentage of GFP expression in transduced cells. Figure 4C shows a graph of GFP expression levels in Vero cells transduced at different dilutions, which shows that the increase in expression at a dilution of 1 was statistically significant compared to other viral dilutions. It is possible that the low transduction efficiency of lentiviruses in Vero cells compared to other cells is due to the inhibitory activity of TRIM5 protein isoforms in the cell.

### Comparison of transfection methods for achieving the highest transfection efficiency in Vero cells

**Fig. 5:**
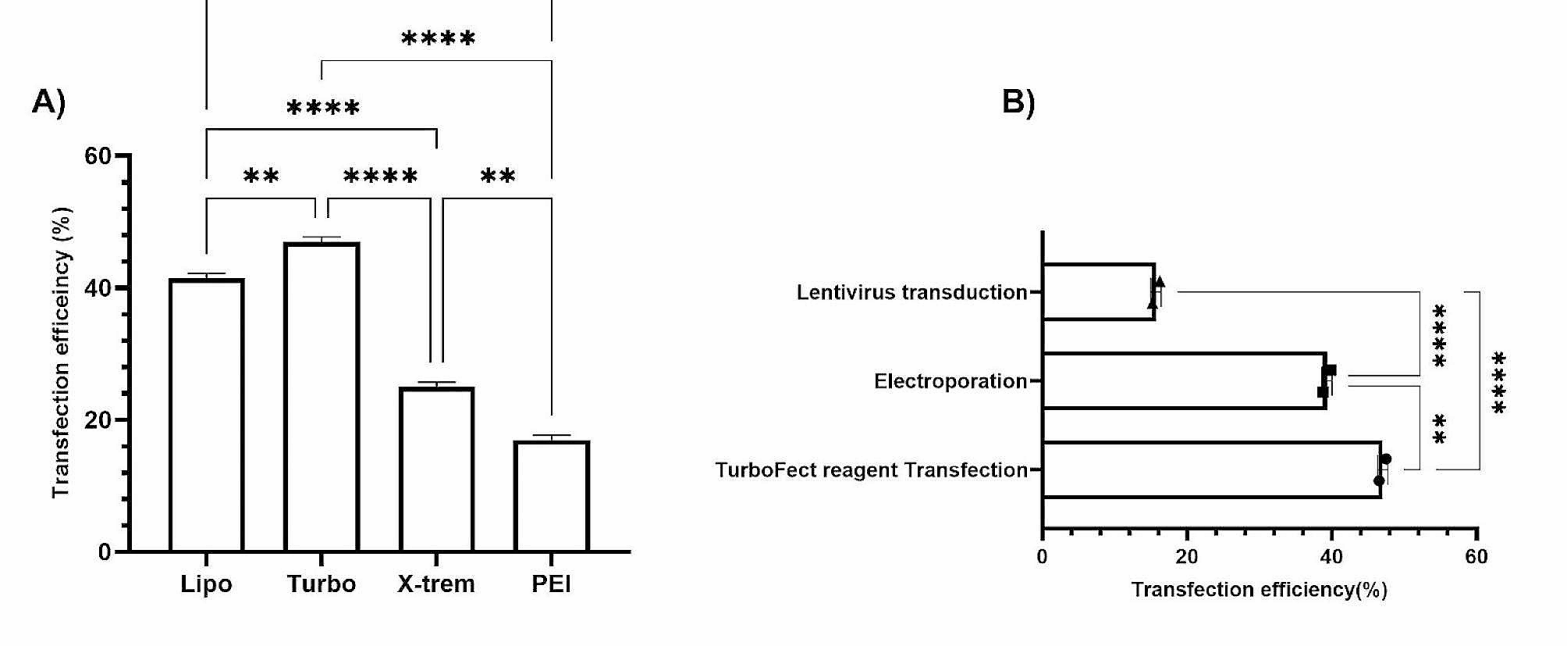
Comparison of all transfection methods. Comparison of transfection with chemical reagents showed that TurboFect had the highest transfection efficiency among other reagents in Vero cells **(A)**. A comparison of all transfection methods showed that the use of chemical reagents, especially TurboFect, is still the most successful method for transferring DNA to Vero cells **(B)**. (*p* ≤ 0.05 *, *p* ≤ 0.01**, *p* ≤ 0.001***, *p* ≤ 0.0001****)

Different transfection methods for assessing optimal transfection in the Vero cell line were used. A comparison of transfection methods based on chemical reagents that showed the highest transfection efficiency revealed that transfection with TurboFect™ resulted in a statistically significant increase in transfection efficiency compared to other methods (*p* ≤ 0.05), and PEI MAX® had a lower transfection efficiency rate (Fig. 5A). As shown in Fig. 4A, other chemical transfection reagents also showed statistically significant differences in transfection efficiency; however, Turbofect had the highest efficiency. Figure 5B shows a comparison between all transfection methods, including lentivirus transduction, electroporation, and TurboFect™ (which is a better chemical transfection method). The results revealed that the TurboFect™ transfection reagent method had the highest transfection efficiency compared with other methods. These results suggest that TurboFect™ has high potential for efficient DNA transfer to Vero cells.

## Discussion

Molecular biology research often involves the introduction of nucleic acids into eukaryotic cells through transfection, which requires appropriate methods depending on cell type and research objectives. Each method has varying levels of transfection efficiency and cell toxicity [14]. However, the ideal method should exhibit a high transfection efficiency with minimal toxicity. Optimization is often necessary to determine optimal transfection conditions [15]. The Vero cell line plays a crucial role in vaccine research; however, an optimized transfection protocol for this cell type is currently lacking. Several factors must be addressed to achieve optimal transfection, including intracellular uptake, endosome acidification, autophagy, immune sensing pathways, and nuclear entry [16]. Numerous factors can influence transfection outcomes. Our focus was to introduce an optimal transfection method for the Vero cell line, which involved careful selection and fine-tuning of the transfection reagent as well as the development of an optimized protocol. In addition, factors such as cell density, incubation time, and the reagent-to-DNA ratio should be considered. Systematically evaluating and adjusting these variables can significantly improve the transfection efficiency in Vero cells, resulting in more reliable and reproducible experimental results [17].

The success of non-viral DNA delivery methods relies on various factors, including the ability of DNA to evade lysosomal degradation, efficient nuclear translocation, decreased cytotoxicity, better control of molecular composition, flexibility in gene size, and lower immunogenicity compared to viral analogs [18]. Studies have shown that lysosomal degradation can be overcome through strategies such as the proton sponge effect in certain cationic polymers, such as PEI. However, efficient nuclear targeting remains a significant challenge during the transfection process. Transfection outcomes can be significantly influenced by factors, such as cell membrane composition and pH, as highlighted by Kim and Eberwine [5].

In this study, we investigated the optimal transfection conditions for the Vero cell line using various chemical transfection agents, including Lipofectamine™ 2000, TurboFect™, X-tremeGENE™ 9, and PEI MAX®. We tested different ratios of DNA and chemical transfection reagents to determine the most effective combination. Our results showed that using 1 µg of each plasmid with 4 µL of TurboFect™ in 6 × 10^4^ cells achieved the highest transfection efficiency (46.5%) and cell viability (94%) compared to other reagents, such as Lipofectamine™ 2000 (42% transfection efficiency and 94% cell viability).

By determining the optimal ratio of reagent to DNA, researchers can enhance the success of transfection experiments while minimizing any negative effects on cell viability. TurboFect™ is a cationic polymer that forms complexes with nucleic acids, thereby facilitating cell attachment, internalization, and endosomal escape. It is particularly suitable for transecting primary cells, difficult-to-transfect cells, and other cell types [19]. Lipofectamine is a cation-lipid-based transfection reagent that forms liposomes to deliver nucleic acids to cells. The composition and structure of liposomes can affect the efficiency and effectiveness of transfection experiments [20].

TurboFect™ has a lower toxicity than Lipofectamine, which contributes to its higher transfection efficiency and cell viability. Choosing TurboFect™ allowed researchers to achieve a balance between effective transfection and high cell viability in Vero cells. In a similar study, CHO-K1 and SH-SY5Y cell lines were transfected with chemical transfection reagents including Lipofectamine 2000, TurboFect 8.0, and ExGen 500. The results showed that transfection efficiency with TurboFect reagent was significantly higher in SH-SY5Y cells compared to the other two types of chemical transfection reagents, and was approximately the same in CHO-K1 cells. The results of this study are consistent with the results of our study and may indicate the success of TurboFect in optimal DNA delivery in other cell lines [21]. We observed a lower transfection efficiency with X-tremeGENE™ 9 and PEI MAX®. PEI, a cationic polymer, exhibited higher cytotoxicity. It can induce toxicity by depolarizing mitochondria and stimulating immune responses. High-molecular-weight PEI (HMW PEI) can form stable polyplexes, but its non-cleavable structure increases its cytotoxicity [22]. PEI– DNA complexes can activate genes involved in cellular responses including apoptosis, stress responses, and oncogenesis [23].

To reduce the cytotoxicity of PEI, researchers can use degradable disulfide-containing polymers, which increase the disassembly rate of the complexes and reduce their binding affinity with intracellular membranes [24]. In addition, free cationic PEI chains embedded in the cell membrane can enhance transfection efficiency by destabilizing the endosomal membrane and hindering its fusion with lysosomes [25].

Overall, our study highlights the importance of selecting an appropriate transfection reagent and the ratio of reagent to DNA to optimize transfection efficiency and maintain cell viability in Vero cells.

Electroporation transfection is a physical method used to introduce external substances into the cells. It involves subjecting cell membranes to high-voltage pulses, which creates tiny pores that allow for the insertion of exogenous genes [26]. Several factors influence the transfection efficiency and cell viability during electroporation. These factors include the electric field strength (voltage), pulse interval (capacitance flow rate), temperature, buffer composition, cell state and volume, and the concentration and structure of foreign genes [27]. Among these factors, electric field strength (voltage) and buffer composition are particularly important in determining transfection efficiency.

In this study, we aimed to identify the optimal electroporation conditions by testing three different electric field intensities (200, 300, and 400 V) and three different buffers with varying ingredients on Vero cell lines. The results showed that all three buffers exhibited the highest transfection efficiency at 300 V, with an electric pulse interval of 850 µF in square waves at all voltages. The cells demonstrated varying levels of adaptation to both electric field intensity and pulse interval. If the voltage is below optimal levels or the electroporation conditions are inadequate for a specific cell strain, no changes occur in the cell membranes, hindering the entry of exogenous genes and reducing the transfection efficiency [28]. Conversely, excessive voltage leads to irreversible cell damage, which significantly affects both cell survival and transfection efficiency [29]. In a similar study investigating the optimal conditions for electroporation of skeletal muscle satellite cells, it was demonstrated that increasing voltage intensity resulted in a decrease in electroporation efficiency and cell viability. DNA dosage was also found to have a significant impact on electroporation success. The optimal voltage at which the highest electroporation efficiency and cell viability were observed varied depending on the cell type, highlighting the importance of determining the optimal electroporation conditions for each cell type [30].

In addition, the results demonstrated that Ebuffer 3 exhibited higher transfection efficiency (38.6%) than the other buffers, and this increase was statistically significant. The composition of the buffer significantly influenced the transfection efficiency. Using RPMI-1640 medium as a shock buffer simplifies this process and reduces cell damage and death after transfection. RPMI-1640 without serum and antibiotics showed higher cell survival after shock than other buffers [10]. Transfection and viability remained unaffected when RPMI-1640 was used as the transfection buffer, even for cells cultured in different media [31].

However, under optimal transfection conditions, the cell viability after electroporation was 56%, which was significantly lower than that of the control. One notable limitation of the electroporation transfection technique is its inherent cellular toxicity, which can range from 50 to 90%. In general, electroporation maintains viability within the range of 30–40% and can be further optimized to maximize transfection efficiency.

HIV-1 lentiviral vectors are commonly used for efficient gene delivery and long-term genetic modification in various cell types, including dividing and non-dividing cells [32]. These vectors offer high titers and low risk of generating replication-competent retroviruses, making them a safe and powerful tool for gene transfer [33]. However, our research indicates that lentivirus particles have a low transduction efficiency in Vero cells, even at high titers, with a reported maximum efficiency of 20%. These findings align with those of previous studies, suggesting that the low transduction efficiency in Vero cells may be due to post-entry restrictions against HIV-1. In particular, innate cellular responses in Vero cells can impede the uncrating process of the virion capsid structure, primarily through the activity of TRIM5 protein isoforms, which possess a defense mechanism against the virus known as ubiquitin ligase activity [34]. Based on these findings, HIV-1 lentiviral vectors are not suitable for efficient transduction of Vero cells. Therefore, alternative lentiviruses derived from different viruses or alternative transfection methods should be considered to achieve optimal and efficient gene transfer into Vero cells [35].

Additionally, we compared various transfection methods to identify the optimal conditions for transfection in Vero cells. The results demonstrated that TurboFect™, a chemical transfection reagent, exhibited the highest transfection efficiency in Vero cells. Non-viral delivery methods, such as chemical transfection, offer advantages over viral vector delivery, including reduced immunogenicity and a lower risk of insertional mutagenesis [36]. Statistically, TurboFect™ significantly outperformed other chemical transfection methods such as electroporation and transduction using lentiviral vectors. Electroporation, although effective for gene delivery, resulted in a high rate of cell death, which negatively affected transfection efficiency. In contrast, transduction using lentiviral vector-based HIV-1 showed low efficiency in Vero cells because of the presence of an intracellular inhibitor for HIV-1 integration [37]. It can be presumed that the high efficiency of TurboFect™ in creating optimal conditions in Vero cells is attributed to the formation of liposome particles, their successful transfer through the lipid membrane, evasion from degradation by lysosomes, and efficient nuclear translocation.

## Conclusions

Efficient transfection of DNA into cells is a valuable tool in molecular biology for applications such as gene therapy, recombinant virus production, and vaccine generation. Owing to their unique characteristics, Vero cells have been used in many studies, including vaccination and virology. Therefore, it would be very valuable to introduce a transfection method with the highest DNA transfer efficiency. Our research findings demonstrate a transfection efficiency of 46.5% when using 1 µg of DNA with 4 µL of TurboFect™ in 6 × 10^4^ cells, which is the highest transfection rate in this cell line. Other transfection methods, such as electroporation, did not yield good results because of high cell mortality. In addition, transduction of lentivectors based on HIV-1 virus inhibits replication of HIV-1 lentivectors in the Vero cell line.

## Data Availability

All data generated or analysed during this study are included in this published article.

## Abbreviations

DNase II: deoxyribonuclease II
DMEM: Dulbecco’s modified Eagle’s medium
CMV: cytomegalovirus
GFP: green fluorescent protein
TU/ml: Transducing units in 1 ml
PEI: Polyethylenimine
HIV-1: human immunodeficiency virus-1
